# Treatment Strategies for Ventricular Septal Rupture After Myocardial Infarction: A Single-Center Experience

**DOI:** 10.3389/fcvm.2022.843625

**Published:** 2022-02-22

**Authors:** Dongliang Ma, Zhibiao Zhang, Shunye Zhang, Zhongchao Wang, Gang Zhang, Chongjun Wang, Jicheng Xi

**Affiliations:** ^1^Department of Cardiovascular Surgery, Shanxi Provincial Cardiovascular Hospital, Shanxi Provincial Institute of Cardiovascular Diseases, Taiyuan, China; ^2^Department of Cardiology, Shanxi Provincial Cardiovascular Hospital, Shanxi Provincial Institute of Cardiovascular Diseases, Taiyuan, China; ^3^Department of Anesthesiology, Shanxi Provincial Cardiovascular Hospital, Shanxi Provincial Institute of Cardiovascular Diseases, Taiyuan, China

**Keywords:** acute myocardial infarction, postinfarction ventricular septal rupture, transcatheter closure, transthoracic closure, thoracotomy

## Abstract

**Objective:**

To analyze treatment strategies, prognosis, and related risk factors of patients with postinfarction ventricular septal rupture, as well as the impact of timing of surgical intervention.

**Methods:**

A total of 23 patients diagnosed with postinfarction ventricular septal rupture who were non-selectively admitted to Shanxi Provincial Cardiovascular Hospital between October 2017 and August 2021 were included in this study. The relevant clinical data, operation-related conditions, and follow-up data were summarized for all patients. The Kaplan-Meier method and log-rank test were used for the cumulative incidence of unadjusted mortality in patients with different treatment methods. Multivariate logistic regression was used to evaluate the independent risk factors for in-hospital patient mortality.

**Results:**

The mean age of the study patients was 64.43 ± 7.54 years, 12(52.2%) were females. There was a significant difference in terms of postoperative residual shunt between the surgical and interventional closure groups (5.9 vs. 100%, respectively; *P* < 0.001). The overall in-hospital mortality rate was 21.7%; however, even though the surgical group had a lower mortality rate than the interventional closure group (17.6 vs. 33%, respectively), this difference was not statistically significant (*P* = 0.576). Univariate analysis showed that in-hospital survival group patients were significantly younger than in-hospital death group patients (62.50 ± 6.53 vs. 71.40 ± 7.37 years, respectively; *P* = 0.016), and that women had a significantly higher in-hospital mortality rate than men (*P* = 0.037). The average postoperative follow-up time was 18.11 ± 13.92 months; as of the end of the study all 14 patients in the surgical group were alive, Two out of four patients survived and two patients died after interventional closure. Univariate analysis showed that interventional closure was a risk factor for long-term death (*P* < 0.05).

**Conclusion:**

Surgical operation is the most effective treatment for patients with postinfarction ventricular septal rupture; however, the best timing of the operation should be based on the patient's condition and comprehensively determined through real-time evaluation and monitoring. We believe that delaying the operation time as much as possible when the patient's condition permits can reduce postoperative mortality. Interventional closure can be used as a supplementary or bridge treatment for surgical procedures.

## Introduction

Postinfarction ventricular septal rupture (PIVSR) is a complication with a low incidence but extremely high mortality. PIVSR can occur 1–14 days after acute myocardial infarction (AMI), with two peak time periods at 24 h and 3–5 days after myocardial infarction (1–3). With the advent of the reperfusion era, the incidence of PIVSR has decreased from 1 to 3% to the current 0.2–0.5% (4, 5). Although there are various treatment methods available such as cardiac assist devices, surgical procedures, and interventional closures, the mortality rate of PIVSR is still high. Studies have shown (6) that the survival rate of PIVSR without surgery after 1 month is <10%, and that surgery can significantly improve the prognosis of these patients. Therefore, surgical treatments are necessary, but the post-surgical mortality rate is still high, and there are still many controversies about the optimum surgical timing. The American College of Cardiology Foundation/American Heart Association (ACCF/AHA) guidelines propose that patients with PIVSR should be operated immediately regardless of their hemodynamic status (7), but the risk of fragile myocardial tissue bleeding and residual shunt after emergency surgery is present; therefore, an increasing number of scholars believe that the timing of the operation should be delayed when the patient's condition permits. The 2017 European Heart Association (EHA) guidelines for the management of acute myocardial infarction also recommend that delayed surgical treatment be considered for patients with PIVSR and stable hemodynamics after active treatment (8).

Percutaneous ventricular septal rupture closure, an interventional transcatheter closure (TCC) approach for PIVSR, was first reported in 1988 by Locki et al. (9). The procedure has developed over the years to become a less invasive treatment alternative, providing a quicker and less traumatic recovery. Even if the ventricular septal rupturet (VSR) cannot be completely blocked, TCC can promote hemodynamic stability and can be used as bridge therapy until later, more invasive, surgical repair can be performed (10). However, the determination of whether TCC or open chest surgery is the best choice for patients with PIVSR requires further research and discussion. This article systematically reviewed the treatment experience of 23 patients with PIVSR in our hospital. We applied risk factor analysis to determine the potential best treatment strategy and the choice of treatment timing to improve the poor prognosis associated with PIVSR.

## Materials and Methods

### General Data

The study was approved by the Human Research Ethics Committee of the Shanxi Cardiovascular Hospital. Being a retrospective study, individual patient informed consent was waived. A total of 23 patients diagnosed with AMI and VSR who were non-selectively admitted to Shanxi Cardiovascular Hospital between October 2017 and August 2021 were included in this study. The enrolled cases, which exclude VSR secondary to the presence of congenital heart disease or resulting from a previous surgical procedure or by trauma or other reasons and those receiving medical treatment. General clinical data, intraoperative data, and relevant postoperative data were collected for all patients and followed up after discharge. This was a consecutive series of all patients and primary data were extracted from the electronic medical or archived records by trained staff. Inclusion criteria were the following: a diagnosis of MI with VSR determined by a clear history of chest pain before surgery, electrocardiograph changes and elevated myocardial enzymes, a systolic murmur heard in the third to fourth intercostal space on the left sternal border, and the presence of a ventricular left-to-right shunt identified on echocardiography. All patients underwent coronary angiography before surgery to confirm the presence of coronary artery disease, and percutaneous coronary intervention was performed at the same time if necessary. The definition of cardiogenic shock (CS) was based on clinical and hemodynamic criteria, including hypotension ([systolic blood pressure (SBP) <90 mmHg] over 30 min or supportive measures required to maintain SBP > 90 mmHg) and evidence of end-organ hypoperfusion (11). Renal insufficiency was defined as serum creatinine levels higher than 120.2 μmol/L or the need for renal replacement therapy (12). The patients were divided into two groups according to the treatment method performed: surgical group (*n* = 17) and interventional closure group (*n* = 6); additionally, the patients were divided into two groups according to outcome, survival group (*n* = 18), and death group (*n* = 5).

### Treatment Methods

Among the 23 study patients, 17 underwent surgical repair, eight of which required simultaneous coronary artery bypass grafting (CABG), and six underwent interventional closure, of which four were treated with TCC and two were treated with transthoracic closure.

#### Surgery

A median sternum incision was used, cardiopulmonary bypass (CPB) was established as per routine, and antegrade perfusion was used to arrest the heart. Subsequently, the following steps were performed: (1) The great saphenous vein or left internal mammary artery was selected as the graft vessel for CABG according to the condition of the coronary artery disease. (2) If a ventricular aneurysm was present it was resected simultaneously by opening the left ventricle about 1–2 cm parallel to the anterior or posterior descending branch. The left ventricle was closed by a circular suture or Dor method and sandwich method. (3) If mitral or tricuspid valve disease was present, valve replacement or plasty was performed simultaneously. In our study, two patients underwent simultaneous valve surgery, and one underwent mitral valve replacement and tricuspid annular implantation to form a tricuspid valve; Another case of tricuspid valve formation using the kay method. (4) The ventricular septal rupture was repaired by trimming the necrotic myocardium and using a single patch for tension-free repair.

#### Interventional Closure

##### TCC

Under local anesthesia, the femoral artery, femoral vein or internal jugular vein were punctured, a catheter was placed in the femoral sheath, and left ventriculography was performed to identify the location and size of the ventricular septal perforation. The angiographic catheter was then passed through to the left ventricle, rotated and lifted toward the perforation at the interventricular septum, passed through the perforation and right ventricle to the pulmonary artery. A multipurpose catheter (Shanghai Shape Memory Alloy Co., Ltd, Shanghai, China) was placed in the femoral vein or internal jugular vein sheath, the loach wire was snared from the venous sheath and subsequently exteriorized to establish a complete arteriovenous loop. An appropriate delivery system (Shanghai Shape Memory Alloy Co., Ltd, Shanghai, China) was placed through the perforation to the apex through the venous side, the inner sheath was withdrawn, and the loach was retained. A VSD occlude (Shanghai Shape Memory Alloy Co., Ltd, Shanghai, China) loaded on a delivery rod was passed through the outer sheath, and the lateral disc of the occluder was first released. The waist and the right ventricular lateral disc of the occluder were released through the perforation, and the occlusion umbrella was completely released after adequate position was determined with left ventriculography and esophageal ultrasound.

##### Transthoracic Interventional Closure Treatment

The transthoracic interventional closure procedure was performed as follows: (1) Median thoracotomy was created under general anesthesia. (2) The purse string was sutured on the right ventricular surface. (3) A sheath (Shanghai Shape Memory Alloy Co., Ltd, Shanghai, China) was used to puncture through the sutured area. (4) A guide wire was placed along the sheath to passed through the ventricular septal rupture to the left ventricle and the sheath was removed. (5) An appropriate delivery system (Shanghai Shape Memory Alloy Co., Ltd, Shanghai, China) was advanced over the guide wire and passed through the ventricular septal rupture, and the guide wire and inner sheath were removed. The other steps to occlude the VSR are the same as those of conventional percutaneous intervention.

### Statistical Methods

All data were statistically analyzed using SPSS 22.0(IBM, Armonk, NY, USA). Normally distributed data are expressed as mean ± standard deviation, and the *t*-test was used for comparison between groups. Non-normal data are expressed as median (interquartile range), and a non-parametric test was used. Count data is expressed as frequency and percentage, and the χ2 test (Fisher exact probability method) was used for comparison between groups. Multivariate analysis with a logistic regression model was used to assess independent risk factors for in-hospital mortality; meanwhile, the Kaplan-Meier method was used to estimate the cumulative incidence of unadjusted mortality in patients who underwent different treatment methods, and the log-rank test was used for comparison. Multivariate Cox proportional hazards regression was used to assess the hazard ratio (HR) and 95% confidence interval (CI) of the relationship between risk factors and mortality. *P* < 0.05 was considered statistically significant.

## Results

**Figure 1:**
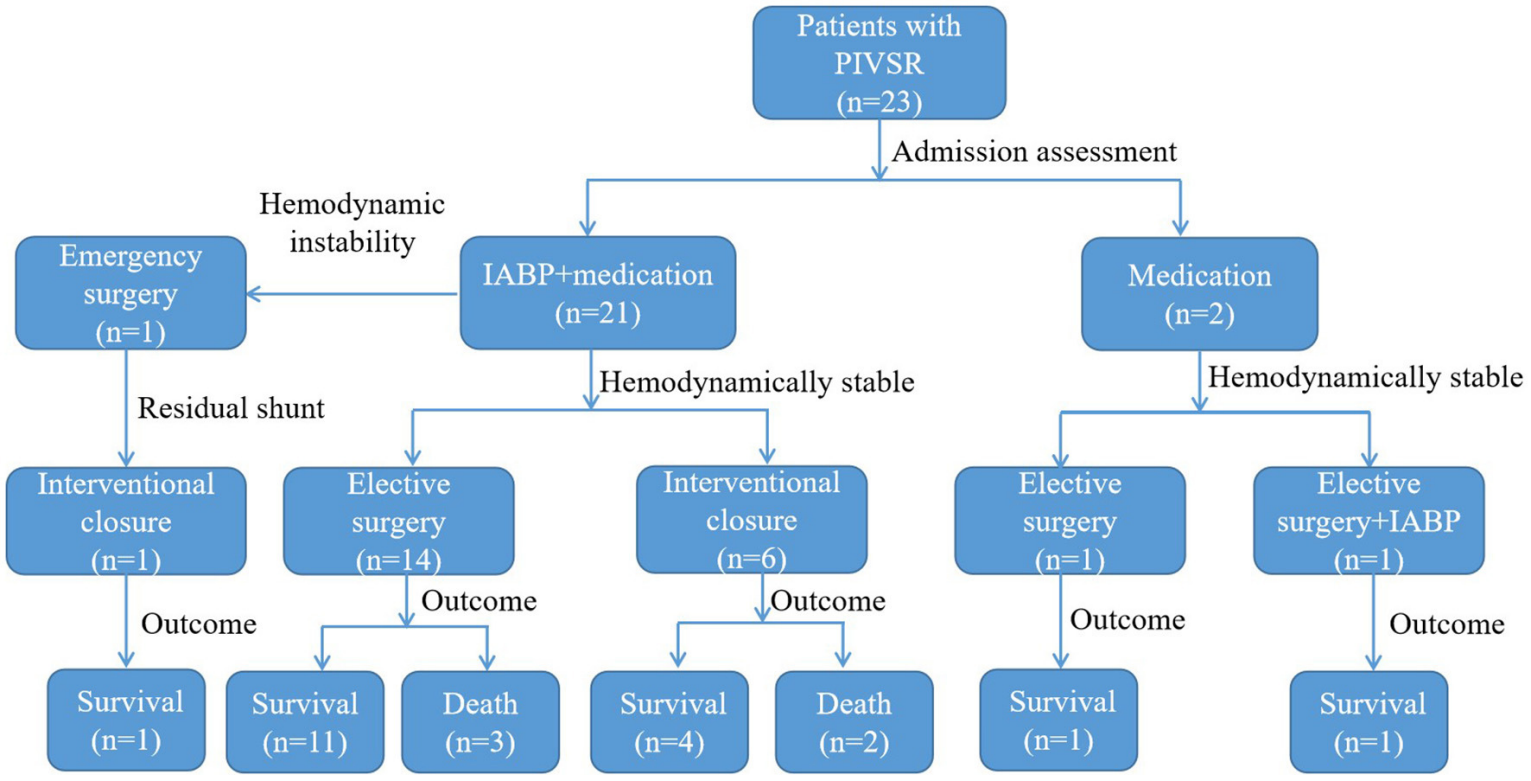
The flow chart in the management and timing of postinfarction ventricular septal rupture.

Atotal of 23 patients were included in this study, with a mean age of 64.43 ± 7.54 years, including 11 males (47.8%, 11/23) and 12 females (52.2%, 12/23). Figure 1 shows the management and time flow for all patients. The median time from the onset of AMI to the diagnosis of VSR was 2 days (interquartile range, 0.83–6 days), and the average time from AMI to surgery was 33.57 ± 10.14 days. One patient (4.3%, 1/23) was operated within 1 week of perforation and 22 patients (95.7%, 22/23) were operated 3 weeks after perforation. The baseline characteristics according to the different treatment methods are shown in Table 1. The preoperative brain natriuretic peptide (BNP) of patients in the interventional closure treatment group was significantly higher than that of patients in the surgical treatment group (7276.50 ± 3468.91 vs. 2872.24 ± 1618.91 ng/L, respectively; *P* = 0.025). A total of seven patients presented a residual shunt, including one patient from the surgery group (5.9%, 1/17) and six patients from the interventional closure group (100%, 6/6) and this difference was statistically significant (*P* < 0.001). The overall in-hospital mortality rate was 21.7% (5/23); however, even though the surgical group had a lower mortality rate than the interventional closure group [17.6% (3/17) vs. 33% (2/6), respectively], this difference was not statistically significant (*P* = 0.576).

**Table 1:** Baseline characteristics of patients with different treatment modalities.

**Characteristics**		**Interventional closure (*n* = 6)**	**Surgical operation (*n* = 17)**	***p-*value**
Age (years)		66.00 ± 7.82	63.88 ± 7.61	0.567
Sex *n* (%)	Male	2 (33.3%)	9 (52.9%)	0.640
	Female	4 (66.7%)	8 (47.1%)	
LVEF (%)		45.33 ± 7.06	47.76 ± 7.09	0.478
LVEDD (mm)		53.50 ± 5.09	52.18 ± 6.00	0.636
AMI to VSR time (days)		1.00 [0.76–4.75]	5.00 [0.79–6.50]	0.343
AMI to surgical time (days)		29.33 ± 8.19	35.06 ± 10.55	0.243
Size of main VSR (mm)		10.00 [7.50–12.00]	10.00 [7.00–20.00]	0.622
VSR location *n* (%)	Anterior	5 (83.3%)	14 (82.4%)	0.730
	Posterior	1 (16.7%)	3 (17.6%)	
BNP (ng/L)		7276.50 ± 3468.91	2872.24 ± 1618.91	**0.025**
Creatinine (μmol/L)		108.00 ± 43.91	94.67 ± 26.07	0.131
ALT (u/L)		24.60 [15.75–156.83]	23.00 [14.50–45.50]	0.674
History of smoking *n* (%)		1 (16.7%)	7 (41.2%)	0.369
Complications *n* (%)				
	Hypertension	4 (66.7%)	8 (47.1%)	0.640
	Hyperlipidemia	2 (33.3%)	2 (11.8%)	0.270
	Diabetes mellitus	3 (50.0%)	4 (23.5%)	0.318
	History of stroke	0 (0.0%)	4 (23.5%)	0.539
	Renal insufficiency	1 (16.7%)	2 (11.8%)	0.616
	Mitral insufficiency	0 (0.0%)	1 (5.9%)	0.739
	Tricuspid insufficiency	0 (0.0%)	3 (17.6%)	0.539
Angiographic data *n* (%)	Negative	0 (0.0%)	1 (5.9%)	0.838
	One-vessel disease	5 (83.3%)	10 (58.8%)	
	Two-vessel disease	0 (0.0%)	3 (17.6%)	
	Three-vessel disease	1 (16.7%)	3 (16.7%)	
Emergency treatment *n* (%)	Negate	6 (100.0%)	16 (94.1%)	0.739
	Right	0 (0.0%)	1 (5.9%)	
Revascularization *n* (%)	–	1 (16.7%)	6 (35.3%)	0.621
	+	5 (83.3%)	11 (64.7%)	
IABP *n* (%)	–	0 (0.0%)	1 (5.9%)	0.739
	+	6 (100.0%)	16 (94.1%)	
Residual shunt *n* (%)	–	0 (0.0%)	16 (94.1%)	**<0.001**
	+	6 (100.0%)	1 (5.9%)	
Outcomes at discharge *n* (%)	Death	2 (33.3%)	3 (17.6%)	0.576
	Survival	4 (66.7%)	14 (82.4%)	

*Data are presented as mean ± standard deviation, mean [range] or number (%)*.

*The en dash (–) denotes absence and the plus sign (+) denotes presence*.

*ALT, alanine aminotransferase; AMI, acute myocardial infarction; BNP, brain natriuretic peptide; IABP, intra-aortic balloon pump; LVEDD, left ventricular end diastolic dimension; LVEF, left ventricular ejection fraction; VSR, ventricular septal rupture; n, number; SD, standard deviation. P values in bold meant significantly different (P < 0.05)*.

Univariate analysis showed that patients in the in-hospital survival group were significantly younger than those in the in-hospital death group (62.50 ± 6.53 vs. 71.40 ± 7.37 years, respectively; *P* = 0.016), and that women had a significantly higher in-hospital mortality rate than men (*P* = 0.037). The baseline characteristics of all patients are shown in Table 2. From the results of univariate analysis, factors with *P* < 0.1 were selected for multivariate analysis and no significant difference was found in regards to renal insufficiency, coronary artery disease, and the presence or absence of revascularization factors. This may be related to the small number of cases, and the details are shown in Table 3.

**Table 2:** Baseline characteristics of hospitalized patients.

**Characteristics**		**In-hospital survival (*n* = 18)**	**In-hospital death (*n* = 5)**	***P*-value**
Age (years)		62.50 ± 6.53	71.40 ± 7.37	**0.016**
Sex *n* (%)	Male	11 (100.0%)	0 (0.0%)	**0.037**
	Female	7 (58.3%)	5 (41.7%)	
LVEF (%)		46.61 ± 7.00	49.00 ± 7.52	0.513
LVEDD (mm)		53.33 ± 5.82	49.60 ± 4.56	0.202
AMI to VSR time (days)		2.00 [0.57–6.00]	6.00 [0.92–10.50]	0.331
AMI to surgical time (days)		32.33 ± 9.31	38.00 ± 12.87	0.279
Size of main VSR (mm)		10.00 [7.00–15.75]	10.00 [7.50–25.00]	0.574
VSR location *n* (%)	Anterior	15 (78.9%)	4 (21.1%)	0.654
	Posterior	3 (75.0%)	1 (25.0%)	
BNP (ng/L)		2829.00 [1914.00–4863.00]	5683.00 [1664.50–10643.00]	0.333
Creatinine (μmol/L)		26.30 [15.23–45.53]	22.00 [16.55–37.55]	0.682
ALT (u/L)		80.65 [66.98–107.50]	85.70 [58.55–105.90]	0.766
Treatment method *n* (%)	Interventional closure	4 (66.7%)	2 (33.3%)	0.576
	Surgical operation	14 (82.4%)	3 (17.6%)	
Hypertension *n* (%)	–	9 (81.8%)	2 (18.2%)	0.545
	+	9 (75.0%)	3 (25.0%)	
Hyperlipidemia *n* (%)	–	15 (78.9%)	4 (21.1%)	0.654
	+	3 (75.0%)	1 (25.0%)	
Diabetes mellitus *n* (%)	–	12 (75.0%)	4 (25.0%)	0.508
	+	6 (85.7%)	1 (14.3%)	
History of smoking *n* (%)	–	10 (66.7%)	5 (33.3%)	0.089
	+	8 (100.0%)	0 (0.0%)	
History of stroke *n* (%)	–	16 (84.2%)	3 (15.8%)	0.194
	+	2 (50.0%)	2 (50.0%)	
Renal insufficiency *n* (%)	–	17 (85.0%)	3 (15.0%)	0.107
	+	1 (33.3%)	2 (66.7%)	
Mitral insufficiency *n* (%)	–	17 (77.3%)	5 (22.7%)	0.783
	+	1 (100.0%)	0 (0.0%)	
Tricuspid insufficiency *n* (%)	–	15 (75.0%)	5 (25.0%)	0.461
	+	3 (100.0%)	0 (0.0%)	
Residual shunt *n* (%)	–	13 (81.3%)	3 (18.7%)	0.621
	+	5 (71.4%)	2 (28.6%)	
IABP *n* (%)	–	1 (100.0%)	0 (0.0%)	0.783
	+	17 (77.3%)	5 (22.7%)	
Revascularization *n* (%)	–	5 (71.4%)	2 (28.6%)	0.621
	+	13 (81.3%)	3 (18.7%)	

*Data are presented as mean ± standard deviation, mean [range] or number (%)*.

*The en dash (–) denotes absence and the plus sign (+) denotes presence*.

*ALT, alanine aminotransferase; AMI, acute myocardial infarction; BNP, brain natriuretic peptide; IABP, intra-aortic balloon pump; LVEDD, left ventricular end diastolic dimension; LVEF, left ventricular ejection fraction; VSR, ventricular septal rupture. P values in bold meant significantly different (P < 0.05)*.

**Table 3:** Logistic regression results of the prognosis of death in hospitalized patients.

**Variable quantity**	**β**	**SE**	**Wald**	**ν**	***p***	**HR**	**HR 95% CI**	
							**Inferior limit**	**Superior limit**
Age	11.236	1525.673	0	1	0.994	75840.294	0	.
Female	70.094	19659.209	0	1	0.997	2.763 ×10^30^	0	.
History of smoking	46.471	21002.050	0	1	0.998	1.521 ×10^20^	0	.
Renal insufficiency	37.640	33900.579	0	1	0.999	2.222 ×10^16^	0	.
Angiographic data								
One-vessel disease	7.974	41349.822	0	1	1.000	0	0	.
Two-vessel disease	77.659	44961.076	0	1	0.999	0	0	.
Three-vessel disease	22.920	51696.798	0	1	1.000	0	0	.
Revascularization	45.099	7903.875	0	1	0.995	0	0	.
Constant quantity	786.803	113059.707	0	1	0.994	0	1	.

*CI, confidence intervals; HR, hazard ratio; SE, standard error*.

All 17 surgical cases were performed under CPB. The average CPB time was 125.94 ± 45.72 min, and the average aortic cross-clamp time was 84.82 ± 32.89 min. Among patients in the surgical group, two underwent simultaneous valve surgery (11.8%, 2/17), 16 underwent simultaneous ventricular aneurysm resection (94.1%, 16/17), and 11 underwent revascularization. A total of 8 patients (47.1%, 8/17) underwent CABG during surgical repair of PIVSR. The postoperative time on ventilator of patients in the death group was longer than those in the survival group, however, this difference was not statistically significant (*P* = 0.386). Details are shown in Table 4.

**Table 4:** Surgical-related data of surgical patients.

**Characteristics**		**Survival (*n* = 14)**	**Death (*n* = 3)**	***P*-value**
CPB time (min)		132.57 ± 43.93	95.00 ± 49.39	0.206
Aortic cross-clamp time (min)		88.21 ± 32.81	69.00 ± 34.66	0.375
Operation time (min)		352.86 ± 79.15	303.33 ± 63.51	0.330
Time of ventilator treatment (h)		68.64 ± 58.17	102.00 ± 62.00	0.386
Concomitant aneurysmectomy *n* (%)	–	1 (100.0%)	0 (0.0%)	0.824
	+	13 (81.3%)	3 (18.8%)	
Concomitant valve surgery *n* (%)	–	12 (80.0%)	3 (20.0%)	0.669
	+	2 (100.0%)	0 (0.0%)	
Re-exploration for bleeding *n* (%)	–	13 (81.2%)	3 (18.8%)	0.824
	+	1 (100.0%)	0 (0.0%)	
Revascularization *n* (%)	–	4 (66.7%)	2 (33.3%)	0.515
	+	10 (90.9%)	1 (9.1%)	

*Data are presented as mean ± standard deviation or number (%)*.

*The en dash (–) denotes absence and the plus sign (+) denotes presence*.

*CPB, cardiopulmonary bypass*.

**Figure 2:**
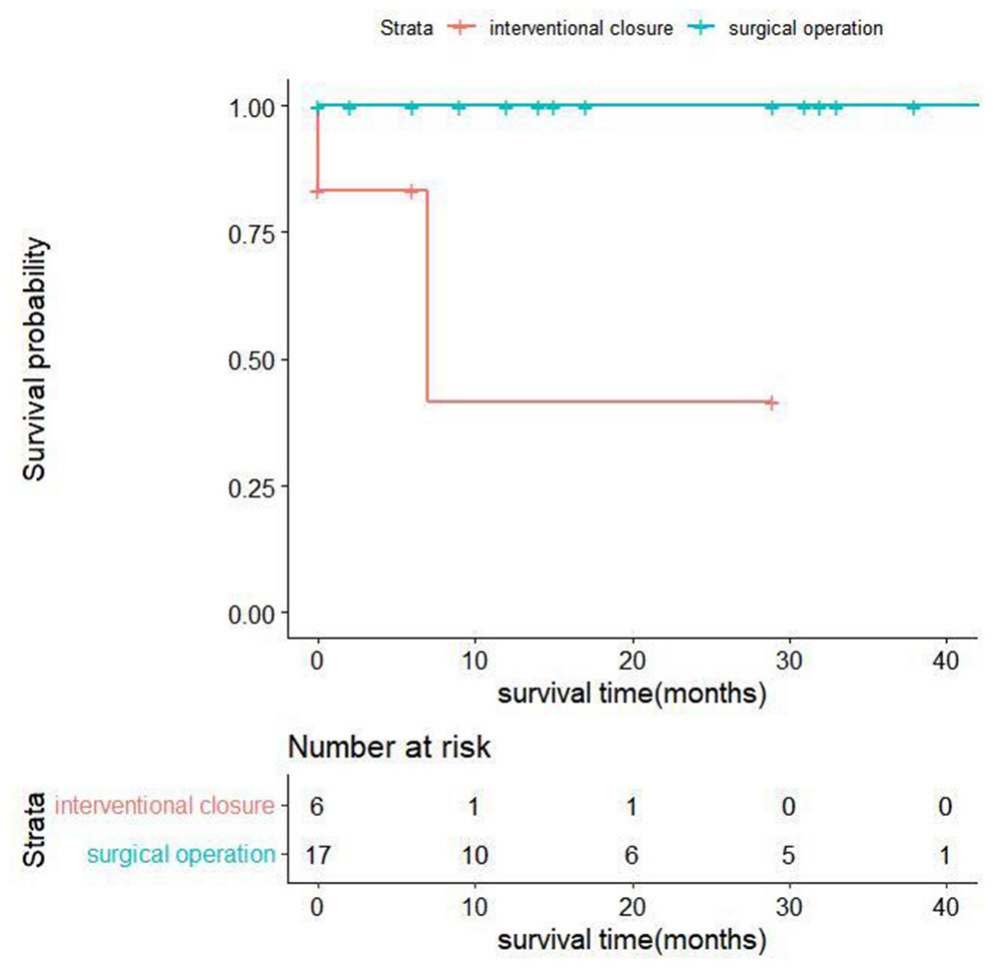
Kaplan-Meier estimates of cumulative event-free survival for long-term mortality.

All discharged patients were followed up, with an average follow-up time of 18.11 ± 13.92 months. From the surgical group, 14 patients were alive at the end of this study, and their cardiac function classifications were all grade II [New York Heart Association (NYHA) classification]. In the interventional closure group, two out of four patients survived; one patient had cardiac function grade II (NYHA grade), and one patient had cardiac function grade III (NYHA grade); two patients died. The baseline characteristics of all follow-up patients are shown in Table 5. The results of univariate analysis showed that the follow-up mortality rate of patients in the interventional closure group was significantly higher than that of patients in the surgical group (*P* < 0.05). Figure 2 also shows the difference in the cumulative long-term survival rate of patients under different treatment methods. Compared with patients treated by surgery, the long-term mortality rate of patients treated with interventional closure was significantly higher. The results of multivariate Cox regression analysis showed that no factors were significantly associated with the death group, which may be related to the small number of cases and short follow-up time, and the details are shown in Table 6.

**Table 5:** Baseline characteristics of discharged patients with follow-up.

**Characteristics**		**Survival (*n* = 16)**	**Death (*n* = 2)**	***P*-value**
Age (years)		62.19 ± 6.87	65.00 ± 1.41	0.581
Sex *n* (%)	Male	10 (90.9%)	1 (9.1%)	0.641
	Female	6 (85.7%)	1 (14.3%)	
LVEF (%)		46.31 ± 6.79	49.00 ± 11.31	0.623
LVEDD (mm)		53.75 ± 5.96	50.00 ± 4.24	0.407
AMI to VSR time (days)		3.28 ± 3.22	7.50 ± 7.78	0.145
AMI to surgical time (days)		32.50 ± 9.73	31.00 ± 7.07	0.837
Size of main VSR (mm)		12.56 ± 6.65	8.00 ± 2.83	0.362
VSR location *n* (%)	Anterior	14 (93.3%)	1 (6.7%)	0.314
	Posterior	2 (66.7%)	1 (33.3%)	
BNP (ng/L)		3419.69 ± 2209.29	3737.00 ± 2491.84	0.852
Creatinine (μmol/L)		100.58 ± 184.30	13.50 ± 6.37	0.525
ALT (u/L)		95.71 ± 35.24	70.70 ± 11.60	0.345
Treatment method *n* (%)	Interventional closure	2 (50.0%)	2 (50.0%)	**0.039**
	Surgical operation	14 (100.0%)	0 (0.0%)	
Hypertension *n* (%)	–	8 (88.9%)	1 (11.1%)	0.765
	+	8 (88.9%)	1 (11.1%)	
Hyperlipidemia *n* (%)	–	14 (93.3%)	1 (6.7%)	0.314
	+	2 (66.7%)	1 (33.3%)	
Diabetes mellitus *n* (%)	–	12 (100.0%)	0 (0.0%)	0.098
	+	4 (66.7%)	2 (33.3%)	
History of smoking *n* (%)	–	8 (80.0%)	2 (20.0%)	0.477
	+	8 (100.0%)	0 (0.0%)	
History of stroke *n* (%)	–	14 (87.5%)	2 (12.5%)	0.784
	+	2 (100.0%)	0 (0.0%)	
Renal insufficiency *n* (%)	–	15 (88.2%)	2 (11.8%)	0.889
	+	1 (100.0%)	0 (0.0%)	
Mitral insufficiency *n* (%)	–	15 (88.2%)	2 (11.8%)	0.889
	+	1 (100.0%)	0 (0.0%)	
Tricuspid insufficiency *n* (%)	–	13 (86.7%)	2 (13.3%)	0.686
	+	3 (100.0%)	0 (0.0%)	
Residual shunt *n* (%)	–	13 (100.0%)	0 (0.0%)	0.065
	+	3 (60.0%)	2 (40.0%)	
IABP *n* (%)	–	1 (100.0%)	0 (0.0%)	0.889
	+	15 (88.2%)	2 (11.8%)	
Revascularization *n* (%)	–	5 (100.0%)	0 (0.0%)	0.510
	+	11 (84.6%)	2 (15.4%)	

*Data are presented as mean ± standard deviation or number (%)*.

*The en dash (–) denotes absence and the plus sign (+) denotes presence*.

*ALT, alanine aminotransferase; AMI, acute myocardial infarction; BNP, brain natriuretic peptide; IABP, intra-aortic balloon pump; LVEDD, left ventricular end diastolic dimension; LVEF, left ventricular ejection fraction; VSR, ventricular septal rupture. P values in bold meant significantly different (P < 0.05)*.

**Table 6:** Risk factors for death at follow-up.

	**β**	**SE**	**Wald**	**ν**	***p***	**HR**	**HR 95% CI**
Age	0.790	1.112	0.504	1	0.478	2.204	0.249–19.502
Surgical operation	−0499	63.031	0.000	1	0.994	0607	0–2.72 ×10^53^
Diabetes mellitus	−0.083	16.181	0.000	1	0.996	0.920	0–5.46 ×10^13^
Residual shunt	11.697	64.379	0.033	1	0.856	120213.94	0–7.57 ×10^59^
Revascularization	−1.05	19.945	0.003	1	0.958	0.350	0–3.32 ×10^16^

*CI, confidence intervals; HR, hazard ratio; SE, standard error*.

## Discussion

### Overall Prognosis of PIVSR

VSR is a rare but highly lethal complication after AMI. The Global Utilization of Streptokinase and T-PA for Occluded Coronary Arteries (GUSTO) trial reported 84 patients who developed PIVSR out of 41,021 patients (0.2%) treated for MI; 34 patients with PIVSR selected for surgical repair had better outcomes than 35 patients treated medically (30-day mortality, 47 vs. 94%) (6). A study of 116 patients with PIVSR conducted at Beijing Anzhen Hospital in China showed that the overall in-hospital mortality rate was 47.4%, while the mortality rate of patients undergoing surgical treatment was 11.7%, and the mortality of patients treated conservatively with medications was 85.7% (13). A retrospective cohort study of 127 patients with PIVSR in the First Affiliated Hospital of Zhengzhou University in China found that the mortality of conservatively treated patients was extremely high. The 30-day mortality rate was 93.6% (73/78), and the long-term mortality rate was 96.2% (75/78); meanwhile, patients who survived early and underwent VSR repair surgery had a good long-term prognosis, which was similar for both percutaneous TCC surgery (25.8%, 8/31) and surgical repair (22.2%, 4/18) (14). Many studies have confirmed that untreated PIVSR has a high mortality rate, so timely surgical treatment is necessary. At present, surgical repair is the gold standard for the treatment of PIVSR. However, the perioperative mortality of VSR surgery has not been effectively improved in recent years (15). Ronco et al. (16) analyzed 475 patients who underwent PIVSR surgical repair in 26 different centers around the world from January 2001 to December 2019 and found that the early mortality rate was 40.4%, and the mortality rate did not improve in the 20 years considered in the study (average 41.7% [32.6–50.0%]). The most common causes of death were low cardiac output syndrome (36.5%) and multiple organ failure (27.6%). In our study the overall in-hospital mortality rate of patients with PIVSR was 21.7% which was lower than the mortality rate in most studies, possibly due to our delayed timing of surgery, and we found that the age of the in-hospital survival group was significantly lower than that of the death group (*P* = 0.016) and the mortality rate for women was significantly higher than men (*P* = 0.037). The causes of death in the surgical group were postoperative intracerebral hemorrhage (*n* = 1), mesenteric embolism after intra-aortic balloon pump (IABP) extraction (*n* = 1), and hyperosmolar coma (*n* = 1); therefore, the cause of death in all three cases was non-cardiac, which suggests the importance of postoperative management. A total of two patients in the interventional closure group died, both because of low cardiac output syndrome, which is consistent with other studies (16).

### Timing of Surgical Treatment

At present, surgery appears to be the best treatment option for patients with PIVSR, since the mortality rate of conservative treatment is very high. The ACCF/AHA guidelines propose that patients with PIVSR should be operated immediately regardless of hemodynamic status, and emergency surgical repair is recommended as a primary indication (7). However, Arnaoutakis et al. (17) analyzed 2876 PIVSR cases from the National Database of the American Society of Thoracic Surgeons from 1999 to 2010 and found that the risk ratios of death from surgical repair of PIVSR were 6.18, 5.53, 4.59, and 2.37 at 6 h, 6–24 h, 1–7 d, and 8–21 d after AMI, respectively, and the risk of death during surgery was significantly reduced when performed >21 days after AMI. In addition, the mortality rates were associated with the timing of the operation as follows: elective operation (13.2%); emergency operation (56.0%); and salvage operation (80.5%). Another study (18) conducted a summary analysis of data collected from the Japanese Adult Cardiovascular Surgery Database for 1,397 patients with PIVSR who underwent surgical repair between 2014 and 2018 and showed that the overall 30-day mortality rate and overall surgical mortality rate were 24.3 and 33.0%, respectively. The surgical mortality rate varied with the surgical situation as follows: elective surgery (15.6%); emergency surgery (30.9%); and emergency/rescue cases (40.6%). Matteucci et al. (19) analyzed 41 studies from January 1998 to February 2020 including a total of 6,361 patients with VSR and found an overall operative mortality rate of 38.2%; however, a statistically significant increase in mortality rate was associated with preoperative/perioperative IABP insertion (OR, 3.48; 95% CI, 3.01–4.02; *P* < 0.001), right ventricular (RV) dysfunction (OR, 2.85; 95% CI, 1.47–5.52; *P* = 0.002), posterior VSR (OR, 1.73; 95% CI, 1.30–2.31; *P* < 0.001) and emergency surgery (OR, 3.79; 95% CI, 2.52–5.72; *P* < 0.001). As these studies show, emergency/rescue surgical procedures are closely related to higher mortality in patient with PIVSR. Because of this, as well as the bleeding problem associated with fragile myocardial tissue in the early stage of VSR and the problem of residual shunt, the timing of surgery for patients with PIVSR is particularly important; however, the optimal timing remains controversial.

The 2017 European Cardiology Society's Acute Myocardial Infarction Management Guidelines recommend that for patients with PIVSR who are hemodynamically stable after active treatment delaying surgical treatment may be considered (8). A 2021 article from the AHA concerning mechanical complications after AMI pointed out that the optimal timing of surgical treatment for patients with PIVSR should be discussed between a cardiac surgeon, cardiologist, and cardiac intensivist, taking into account the severity of CS, organ failure, and risk of coagulopathy attributable to antiplatelet medication; for patients with stable hemodynamics and no respiratory failure, delayed selective surgical repair can be considered (20). Papalexopoulou et al. (21) evaluated six large studies including 3,238 patients who underwent surgery for PIVSR, and the results showed that the in-hospital mortality was 52.4% in patients who underwent surgical repair in the first 3 days to 4 weeks and 7.56% in patients who underwent delayed surgical treatment 1–4 weeks later. Early surgery should be performed promptly if the diameter of the perforation is >15 mm accompanied by significant shunting or hemodynamic deterioration; surgery should be performed immediately if CS occurs; and surgery can be delayed 3–4 weeks in hemodynamically stable patients. Shafiei et al. (22) showed that the optimal time for PIVSR surgery is when the marginal tissue scar formation occurs after VSR maturity. In addition, in a large number of patients, due to the risk of severe heart failure and organ dysfunction, surgery is recommended immediately after diagnosis of PIVSR to prevent further hemodynamic deterioration. In some patients with hemodynamic instability, it is believed that preoperative use of ventricular assist devices, including IABP or extracorporeal membrane oxygenation (ECMO) should be used to delay surgery, thus improving postoperative survival. Based on our retrospective study of 23 patients, we believe that if the patient's condition allows, the operation can be delayed as far as 3–4 weeks after myocardial infarction. Surgical treatment after the formation of scars around the infarcted myocardium can effectively prevent the occurrence of complications such as hemorrhage of the infarcted myocardium, re-enlargement of VSR, or the formation of residual shunt after the infarcted myocardium is reabsorbed. None of our 16 patients who underwent delayed surgery had complications such as residual shunt and bleeding after surgery, which also illustrates the benefits of delayed surgery. However, while waiting for surgery, patients also face the risks of catheter-related infections, pneumonia, atelectasis, venous thrombosis of the lower extremities, and multiple organ dysfunction; some patients may even develop CS that cannot be maintained due to hemodynamic instability and permanently lose the opportunity for surgery. For these patients, active emergency surgery may still be beneficial, but this issue still needs further research and discussion.

Maintaining hemodynamic stability until surgery in patients with VSR is also a great challenge. The development and application of mechanical assistive devices has improved treatment in these cases. Commonly used cardiac assist devices include IABP, ECMO, left ventricular assist device (LVAD), and total artificial heart. The current European Society of Cardiology (ESC) guidelines recommend the use of cardiac assist devices to support treatment before surgical repair and as a bridge to repair perforations (8). Morimura et al. (23) studied eight PIVSR cases treated in our institution between July 2015 and November 2017 and found that all patients were given IABP assistance before surgery; five patients were also placed on ECMO assistance, and all patients successfully avoided emergency surgical treatment. The median time from myocardial infarction to surgery was 7.1 d (interquartile range 3.7–9.9 d), and from PIVSR diagnosis to surgery was 1.9 d (interquartile range 1.3–2.3 d). The final surgical mortality rate was 12.5%, of which one case had mechanical circulatory support-related complications (12.5%), and the 2-year survival rate was 62.5%. Another study (24) analyzed 27 patients with PIVSR and CS treated at their institution between January 2018 and March 2020, and found that emergency surgical repair was avoided in all patients through the use of mechanical assistive devices, with a mean time from MI to VSR repair of 18.85 days, a surgical mortality rate of 11%, and a total mortality rate of 33.3% after one 1 year. Rob et al. (25) found through a case study of PIVSR patients from January 2007 to June 2016 that 28 of 31 patients with PIVSR received IABP assistance, and seven patients with refractory CS received Veno-Arterial Extracorporeal Membrane Oxygenation (V-A ECMO) support preoperatively, with decreased lactate levels (7.9 vs. 1.6 mmol/L, *P* = 0.01), improved mean arterial pressure (64 vs. 83 mmHg, *P* < 0.01), and decreased heart rate (115 vs. 68/min, *P* < 0.01) 24 h after implantation. Their findings showed that early use of V-A ECMO assistance in patients with PIVSR stabilized hemodynamics and had the potential to reverse the fatal process of refractory CS; therefore, early use of V-A ECMO support rather than emergency surgery in patients with PIVSR and CS may suggests the possibility of a paradigm shift in the new management model of these patients, but hemorrhagic complications are also an important limitation of this approach. In our study, 21 of 23 patients were actively given IABP adjuvant therapy after admission to fully assess the condition. One patient was given IABP adjuvant therapy during surgery, and one patient was given active medical treatment for stable disease without IABP treatment. Only one of the patients in our study had severe CS, which was associated with failure of the patient to attend the hospital in the recommended time. In this case emergency surgery was required because IABP and active medications could not improve hemodynamic stability; meanwhile, other patients were successfully delayed until at least 21 days after myocardial infarction, and no death or severe organ dysfunction occurred during the waiting process. Therefore, we believe that in the early stage after perforation, active and effective mechanical assisted circulation not only improves the patient's circulatory conditions, but also helps delay the operation and avoid emergency surgery; of the mechanical auxiliary devices, IABP is a simple circulatory adjuvant device, and it is also the most widely used mechanical auxiliary device; our experience using ECMO is limited. There are also few reports in the relevant literature, and further studies are still needed. It must also be pointed out that possible complications associated with mechanical assistance also require close attention during circulatory support.

### Surgical Methods and Improvements

Surgical repair of PIVSR was first reported by Cooley (26) in 1957, and since then many methods of surgical repair of PIVSR have been introduced, the two most commonly used being the Daggett (27) and David surgical procedures (28). There were also many surgeons trying new surgical techniques. Pacini et al. (29) reported on a triple-layer patch technique used to repair PIVSR in eight patients with good results; there were no cases of residual shunt or cardiac rupture after surgery. Kinoshita et al. (30) successfully performed PIVSR repair for 33 patients using an extended sandwich patch technique, with the 30-day mortality and one-year survival in the early and late groups were 20 and 12.5%, and 58 and 88%, respectively, and providing safe, simple, leak-free repair even in technically demanding acute or post-interval VSR. Nakae et al. (31) recently reported a new infarction exclusion technique which makes use of a new tissue adhesive to place a second patch on the rupture site after a first patch is used to exclude the infarcted myocardium, thereby avoiding suturing of fragile infarcted myocardium, thereby effectively preventing shunt recurrence after VSR. New technologies are proposed and applied to PIVSR patching, but they are often more complex and require very experienced surgical teams; therefore wide acceptance and application require further research and discussion. In our study, all patients in the surgical group underwent a simpler single-layer patch suture technique. Only one patient had a residual shunt after the operation, and this case required emergency surgery due to CS with hemodynamic instability refractory to treatment with drugs and mechanical adjuvant therapy. However, none of the patients who underwent delayed operations had residual shunt or bleeding complications, which may be due to the formation of fibrous scar tissue around the infarcted myocardium, which makes the operation easier and the suture more accurate and firmer. In our experience tension-free suture should be adopted during the operation, and gaskets should be used in places with significant edema to prevent suture from cutting tissue. Intermittent reinforcement with gasketed suture is used for weak areas or areas prone to residual leakage during suturing.

### Development and Application of Interventional Closure Technology

Although surgery is currently the most effective method for the treatment of PIVSR, the high postoperative mortality rate and trauma caused by surgery have led to the acceptance of TCC as a less traumatic alternative and is now widely used in clinical practice. The current indications for TCC mainly include the following: patients who are too old and in poor general condition and so are unsuitable for surgery or who refuse surgical repair; VSR edges have sufficient width to facilitate fixation of the occluder VSR diameter <15 mm, although a new type of occluder that can seal PIVSR with a diameter of 17 mm has been reported (32); PIVSR patients with residual fistula after surgical repair, TCC can be used as the first choice of treatment (33). When performed early, the TCC technique has a high success rate, is less traumatic, and may become a valuable alternative to surgical treatment. However, the mortality rate in the perioperative period is still very high. A number of studies have shown that the mortality rate is between 27.3 and 35% (32, 34, 35), and complications such as heart rupture, residual shunt, occluded umbrella dislocation, hemolysis, stroke, malignant arrhythmia, and bleeding at the puncture site can occur. Another article reported on transthoracic interventional closure technology (36), the other steps to occlude the VSR are the same as those of conventional percutaneous intervention. This surgical method has the advantages of easier crossing of the VSR and better visualization than the TCC technique, avoids cardiopulmonary bypass, and the surgeon does not need to suture the fragile myocardium after infarction. In this study, five patients were treated with TCC, four of which were primary procedures and one was a secondary procedure after developing a residual shunt. Among them, one patient died after surgery. Two patients underwent transthoracic interventional closure treatment, of which one died postoperatively. Compared with the surgical group, the preoperative BNP of the interventional closure treatment group was significantly higher than that of the surgical treatment group (*P* = 0.025), which suggests that the general condition and cardiac function of patients undergoing interventional closure therapy were worse before surgery. This makes sense, since it is one of the reasons why interventional closure therapy was chosen. All six patients had residual shunts to varying degrees after surgery, which was significantly different from the surgical group (*P* < 0.001). The follow-up of all discharged patients and the application of univariate analysis showed that the follow-up mortality rate of interventional closure was also significantly higher than that of surgery (*P* < 0.05). Therefore, we believe that the first choice of treatment for VSR patients is still surgical treatment, and interventional closure treatment can be used as the second choice or as a salvage treatment for residual shunt after surgery.

## Conclusion

In summary, we believe that the overall prognosis of patients with PIVSR is still poor. Surgical treatment is still the best treatment plan and the first choice. Although interventional closure is feasible, it is not the first choice of treatment; However, it should be considered when surgery is deemed inappropriate or patient chooses not to have surgery. Regarding the timing of surgery, we believe that in the event of PIVSR, mechanical assist devices should be used to stabilize the patient's hemodynamics as soon as possible, and the operation time should be delayed as far as possible if the condition permits.

Of course, there are several other limitations to this study. This study is a retrospective study with a small sample size and non-selective data from a single center. There may be data offsets in the study. More in-depth research depends on subsequent large-sample surveys of multiple centers.

## Data Availability Statement

The raw data supporting the conclusions of this article will be made available by the authors, without undue reservation.

## Ethics Statement

The studies involving human participants were reviewed and approved by the Human Research Ethics Committee of the Shanxi Cardiovascular Hospital. Written informed consent for participation was not required for this study in accordance with the national legislation and the institutional requirements.

## Author Contributions

JX, SZ, ZW, and GZ: study design, study analysis, conducted the study, and performed the examinations. DM, ZZ, and CW: collection and assembly of data. DM and JX performed the statistical analysis, wrote the manuscript, and final approval of manuscript. All authors have read and approved the final version of the manuscript.

## Conflict of Interest

The authors declare that the research was conducted in the absence of any commercial or financial relationships that could be construed as a potential conflict of interest.

## Publisher's Note

All claims expressed in this article are solely those of the authors and do not necessarily represent those of their affiliated organizations, or those of the publisher, the editors and the reviewers. Any product that may be evaluated in this article, or claim that may be made by its manufacturer, is not guaranteed or endorsed by the publisher.
